# Supplementary material for: Integrating In Vitro Analytics for Improved Antibody–Drug Conjugate Candidate Selection
Source: Cancers (Basel). 2026 Jan 3;18(1):164. doi: 10.3390/cancers18010164 (PMC12784668; doi:10.3390/cancers18010164)
Supplement: Supplementary file 1 [file cancers-18-00164-s001.zip › Supporting Information_Revised&Confirmed.pdf]

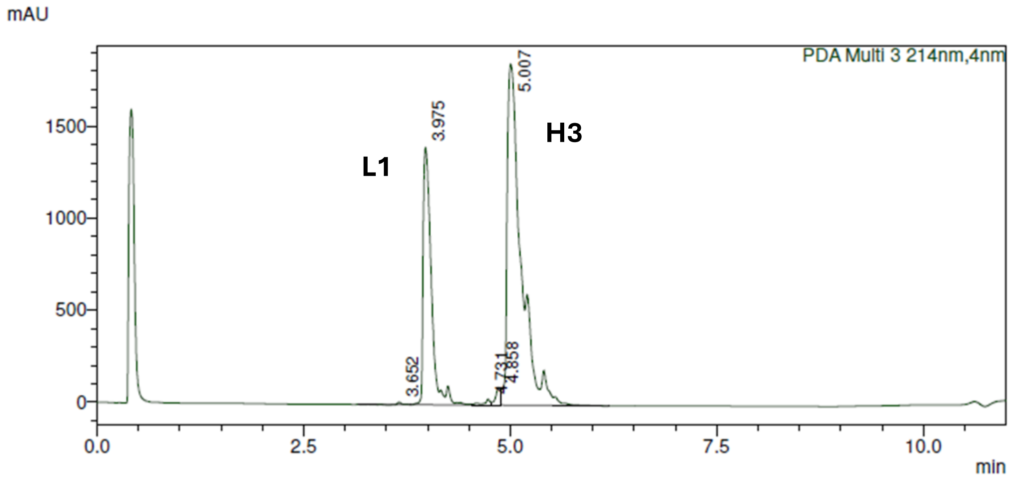

Figure S 1. RP-HPLC chromatogram of ADC with maleimide linker, Her-maleimide.

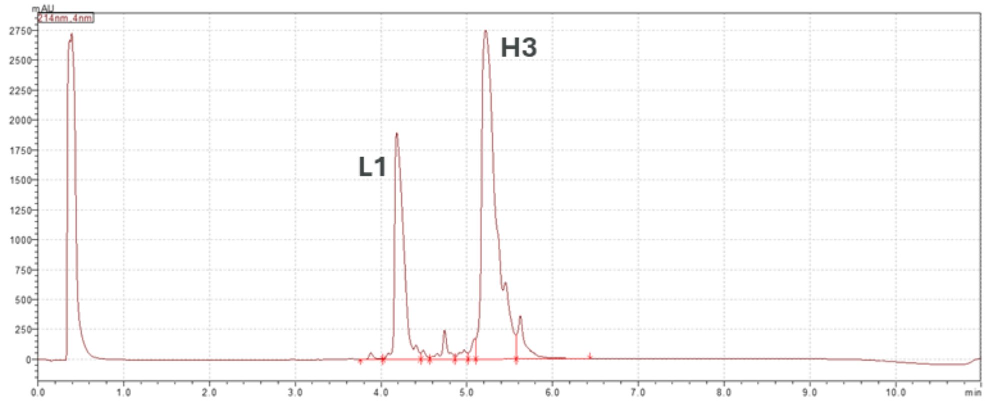

Figure S 2. RP-HPLC chromatogram of ADC with bromoacetamide linker, Her-bromoacatamide.

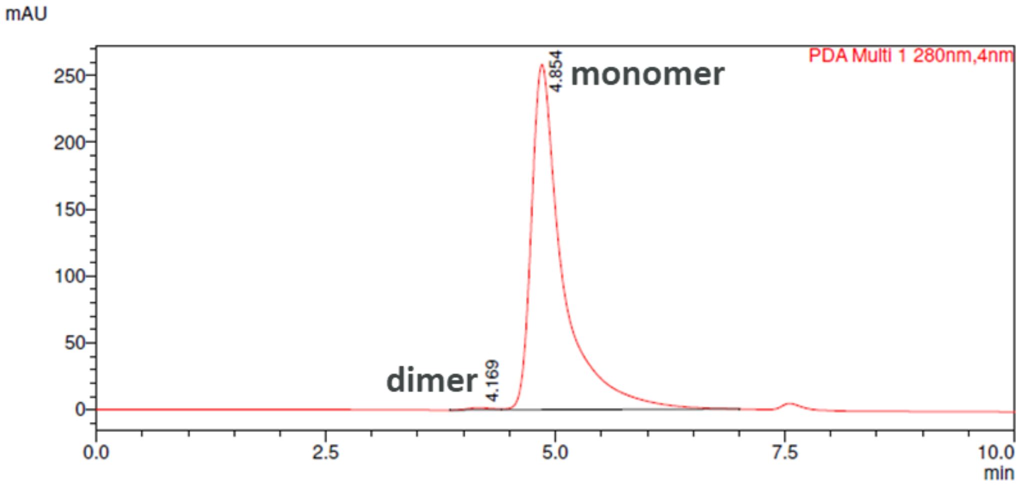

Figure S 3. SEC-HPLC chromatogram of ADC with maleimide linker, Her-maleimide.

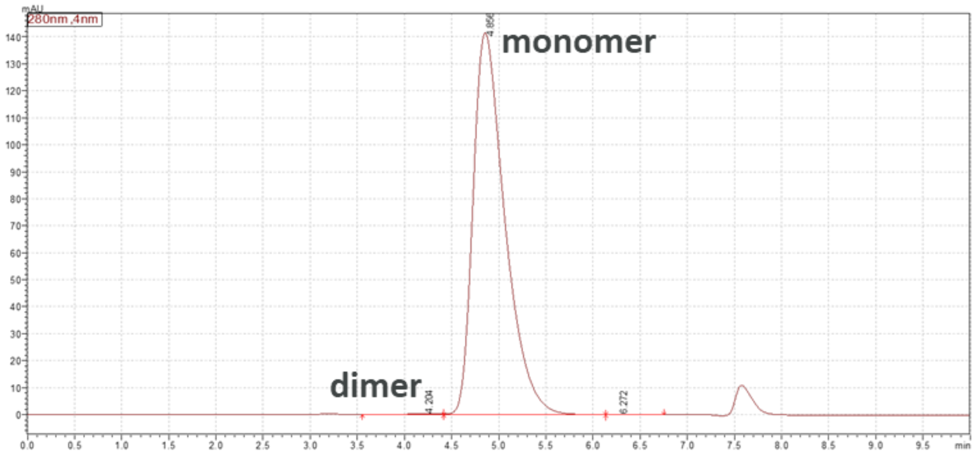

Figure S 4. SEC-HPLC chromatogram of ADC with bromoacetamide linker, Her-bromoacatamide.

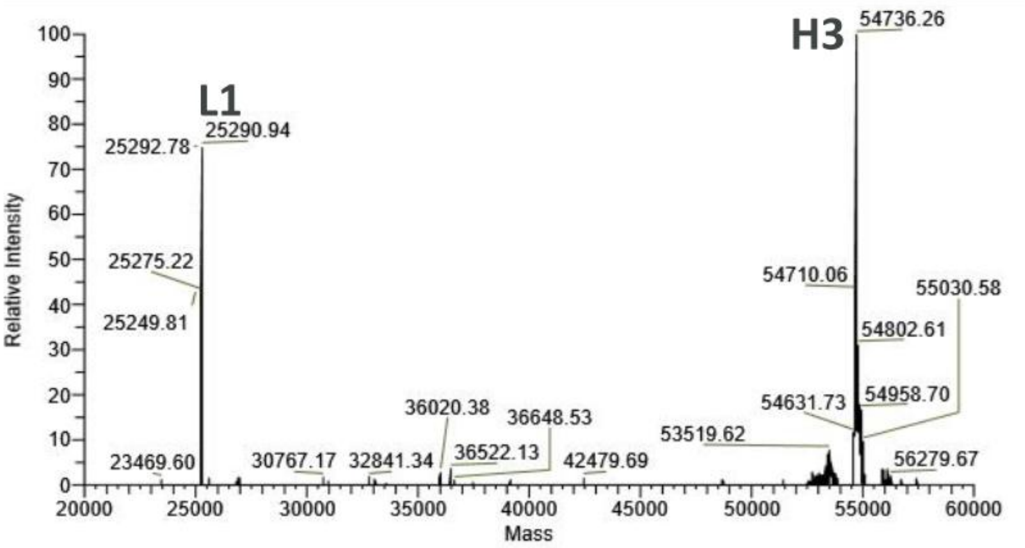

Figure S 5. Deconvoluted spectrum of ADC with maleimide linker, Her-maleimide. Theoretical masses for L1 and H3 are 25292.15652 and 54734.85956 respectively.

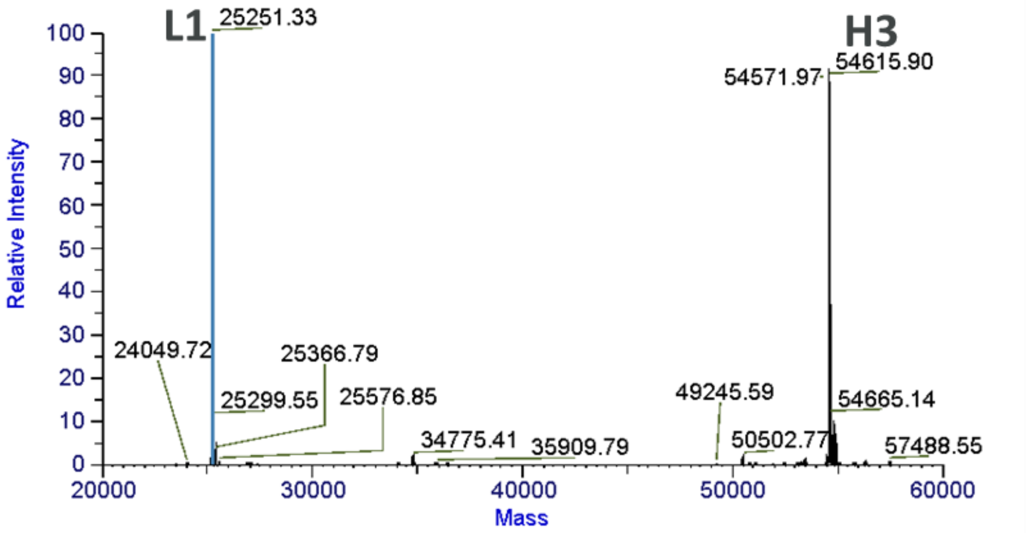

Figure S 6. Deconvoluted spectrum of ADC with bromoacetamide linker, Her-bromoacatamide. Theoretical masses for L1 and H3 are 25252.16493 and 54614.88479 respectively.

1. Power Query inputs and outputs

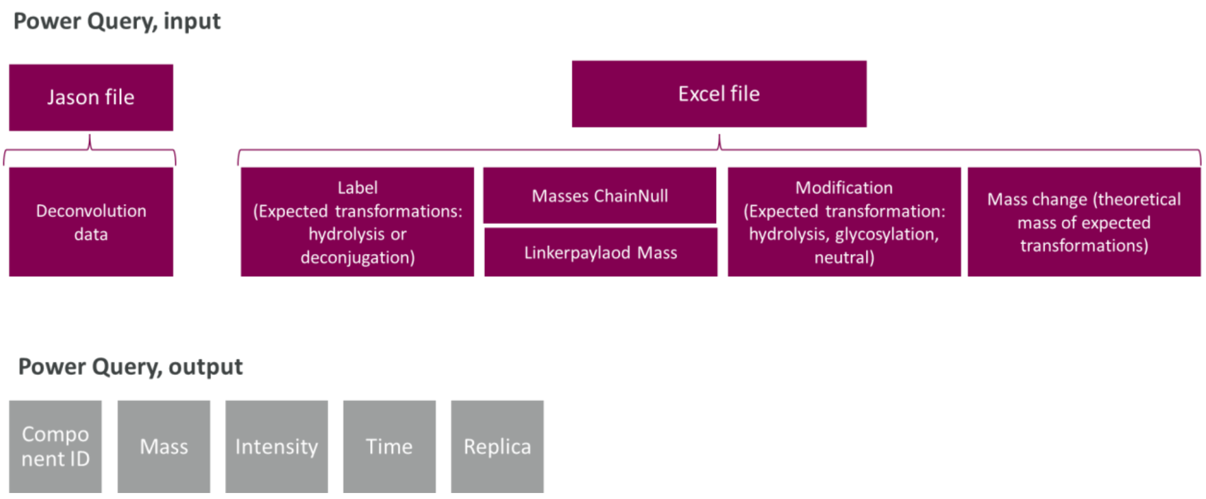

*Scheme S1. **Inputs and outputs from PowerQuery***

2. Identification of species or components

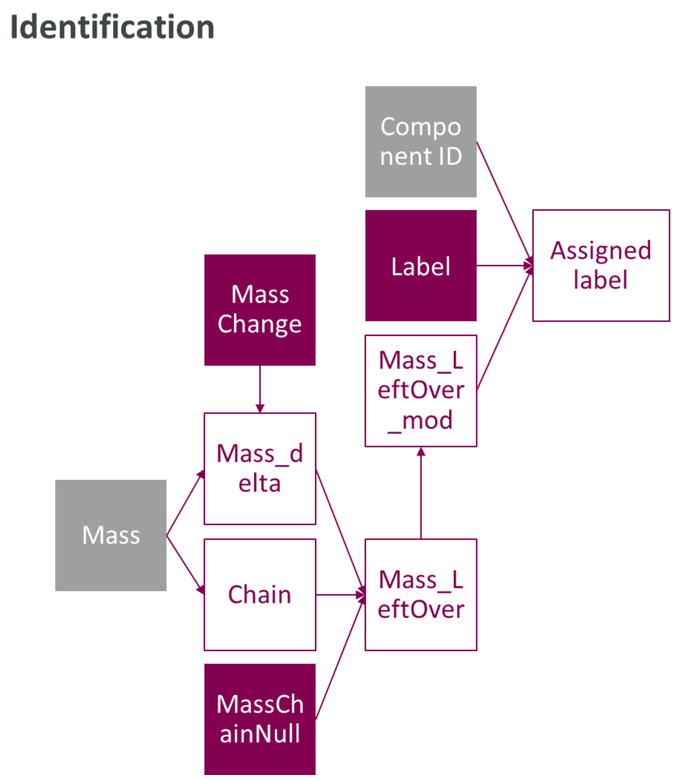

*Scheme S2. **Parameters needed for identification of species***

2.1. "Assigned Label" parameter

```
Assigned_label =
    // Calculate the label
    CALCULATE (
        // Get the first (and only) Label value
        FIRSTNONBLANK ( peaks_ids[Label], 1 ),
```

```
        // Filter the table
        FILTER (
            // All rows in the table
            ALL ( peaks_ids ),
            // Where the ComponentID is the current ComponentID
            peaks_ids[ComponentId] = EARLIER ( peaks_ids[ComponentId] )
            // And the Mass_LeftOver_Mod is below 3
            && peaks_ids[Mass_LeftOver_mod] < 3
        )
    )
```

2.2. “Mass LetfOver mod” parameter

```
Mass_LeftOver_mod = IF(peaks_ids[Mass_LeftOver] < 0, peaks_ids[Mass_LeftOver]*-1,
peaks_ids[Mass_LeftOver])
```

2.3. “Mass LeftOver” parameter

```
Mass_LeftOver = IF(peaks_ids[Chain] = "LC", {peaks_ids[Mass_delta] -
peaks_ids[Mass_LNull]}, {peaks_ids[Mass_delta] - peaks_ids[Mass_HNull]})
```

2.4. “Mass delta” parameter

```
Mass_delta =
//peaks_ids[Mass] - peaks_ids[Mass_change]
//Initialize variables
VAR mass_detected = peaks_ids[Mass]
VAR mass_change = peaks_ids[Mass_change]
// Calculate difference between detected mass and expected modification mass.
VAR mass_delta = mass_detected - mass_change

RETURN mass_delta
```

2.5. “Chain” parameter

```
Chain = IF(peaks_ids[Mass] < 40000, "LC", "HC")
```

2.6. “ResultsResult label” parameter

```
ResultsResult_label =
    // Calculate the Assigned_label value
    CALCULATE (
        // Get the first (and only) Assigned_label value
        FIRSTNONBLANK ( peaks_ids[Assigned_label], 1 ),
        // Filter peaks_ids
        FILTER (
            // All rows in peaks_ids
            ALL ( peaks_ids),
            // Where the ComponentId is the current ComponentId in Results
            peaks_ids[ComponentId] = EARLIER ( Results[ComponentId] )
        )
    )
```

2.7. “Result_DrugLoad” parameter

```
Result_DrugLoad =
VAR this_val = LEFT(Results[ResultsResult_label], 1)
VAR out = IF(ISBLANK(this_val), "-1000", this_val)
RETURN out
```

3. Calculations

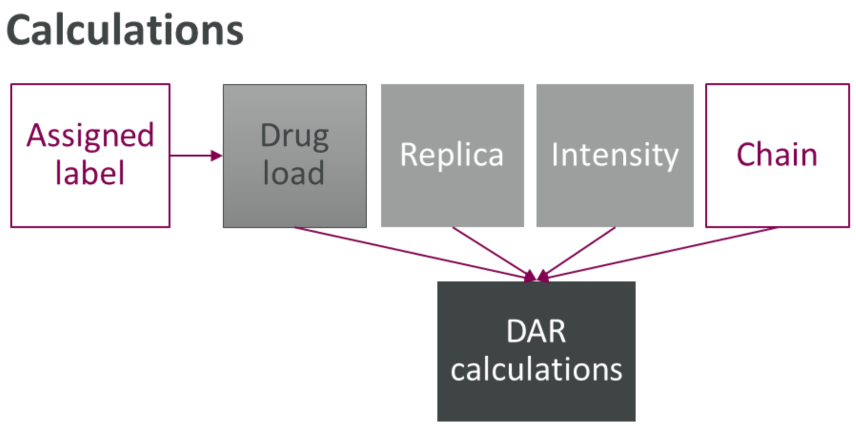

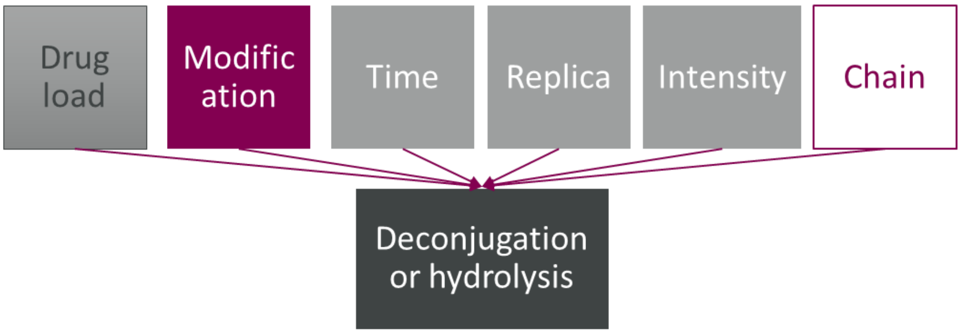

Scheme S3. ***Parameters needed for calculation of DAR value and deconjugation and hydrolysis percentages.***

3.1. DAR calculations

3.1.1. ADC

```
ADC = CALCULATE(
       ([DAR_HCs] + [DAR_LC]),
        'Results'
    )
```

3.1.2. Light chain

```
DAR_LC = 2 * (
AVERAGEX (
    SUMMARIZE (
        Results,
        Results[Replicate],
        "DAR_L",
        // Initialize variables for each replicate
        VAR L0_int = CALCULATE(
            SUM(Results[RawFileResults.Intensity]),
            Results[Chain] = "LC",
            Results[Result_DrugLoad] = 0,
            Results[Replicate] = EARLIER(Results[Replicate])
        )


        VAR light_int = CALCULATE(
            SUM(Results[RawFileResults.Intensity]),
            Results[Chain]= "LC",
            Results[Result_DrugLoad] > 0,
```

```
            Results[Replicate] = EARLIER(Results[Replicate])
        )

        VAR ratio = DIVIDE(light_int, light_int + L0_int, BLANK())

        RETURN
        ratio
    ),
    [DAR_L]
))
```

3.1.3. *Heavy chain*

```
DAR_HC = 2 * (
AVERAGEX (
    SUMMARIZE (
        Results,
        Results[Replicate],
        "DAR_H",
        // Initialize variables for each replicate
        VAR H0_int = CALCULATE(
            SUM(Results[RawFileResults.Intensity]),
            Results[Chain] = "HC",
            Results[Result_DrugLoad] = 0,
            Results[Replicate] = EARLIER(Results[Replicate])
        )

        VAR unique_drug_loads = FILTER(
            VALUES(Results[Result_DrugLoad]),
            Results[Result_DrugLoad] > 0
        )

        VAR heavy_int = CALCULATE(
            SUM(Results[RawFileResults.Intensity]),
            Results[Chain] = "HC",
            Results[Result_DrugLoad] > 0,
            Results[Replicate] = EARLIER(Results[Replicate])
        )

        VAR heavy_total =
            SUMX(
                unique_drug_loads,
                VAR current_drug_load = Results[Result_DrugLoad]
                VAR avg_intensity =
                    CALCULATE(
                        SUM(Results[RawFileResults.Intensity]),
                        Results[Result_DrugLoad] = current_drug_load,
                        Results[Chain] = "HC",
                        Results[Replicate] = EARLIER(Results[Replicate])
                    )
                RETURN
                    avg_intensity * current_drug_load
            )

        VAR ratio = DIVIDE(heavy_total, heavy_int + H0_int, BLANK())

        RETURN
        ratio
    ),
    [DAR_H]
))
```

3.2. Hydrolysis

3.2.1. *Heavy chain*

3.2.1.1. "Hydrolysis" parameter

```
Hhydrolysis = AVERAGEX (
    SUMMARIZE (
        Results,
        Results[Chain],
        Results[Replicate],
        Results[Modification],
        Results[Time],
        "Hall",

        VAR H_all = CALCULATE(
            SUM(Results[RawFileResults.Intensity]),
            Results[Chain] = "HC",
            Results[Result_DrugLoad] > 0,
            Results[Replicate] = EARLIER(Results[Replicate]) &&
            Results[Modification] = "hydrolysis"
        )
       VAR ratio = (H_all)

        RETURN
        ratio
    ),
    [Hall]
)
```

3.2.1.2. "Hneutral" parameter

```
Hneutral = AVERAGEX (
    SUMMARIZE (
        Results,
        Results[Chain],
        Results[Replicate],
        Results[Modification],
        Results[Time],
        "Hall",

        VAR H_all = CALCULATE(
            SUM(Results[RawFileResults.Intensity]),
            Results[Chain] = "HC",
            Results[Result_DrugLoad] > 0,
            Results[Replicate] = EARLIER(Results[Replicate]) &&
            Results[Modification] = "neutral"
        )
       VAR ratio = (H_all)

        RETURN
        ratio
    ),
    [Hall]
)

Hydro_HC = 100*(DIVIDE([Hhydrolysis], ([Hhydrolysis] + + [Hneutral])))
```

3.2.2. *Light chain*

3.2.2.1. "Hydrolysis" parameter

```
Lhydrolysis = AVERAGEX (
    SUMMARIZE (
        Results,
        Results[Chain],
        Results[Replicate],
        Results[Modification],
        Results[Time],
        "Lall",

        VAR L_all = CALCULATE(
            SUM(Results[RawFileResults.Intensity]),
```

```
            Results[Chain] = "LC",
            Results[Result_DrugLoad] > 0,
            Results[Replicate] = EARLIER(Results[Replicate]) &&
            Results[Modification] = "hydrolysis"
        )
        VAR ratio = (L_all)

        RETURN
        ratio
    ),
    [Lall]
)
```

3.2.2.2. "Hydrolysis" parameter

```
Lneutral = AVERAGEX (
    SUMMARIZE (
        Results,
        Results[Chain],
        Results[Replicate],
        Results[Modification],
        Results[Time],
        "Lall",

        VAR L_all = CALCULATE(
            SUM(Results[RawFileResults.Intensity]),
            Results[Chain] = "LC",
            Results[Result_DrugLoad] > 0,
            Results[Replicate] = EARLIER(Results[Replicate]) &&
            Results[Modification] = "neutral"
        )
        VAR ratio = (L_all)

        RETURN
        ratio
    ),
    [Lall]
)

Hydro_LC = 100*(DIVIDE([Lhydrolysis], ([Lhydrolysis] + [Lneutral])))
```

3.3. Deconjugation

3.3.1. *Heavy chain*

3.3.1.1. "TotalDAR HC" parameter

```
TotalDAR_HC =
//Initialize variables
VAR H0_int = CALCULATE( SUM(Results[RawFileResults.Intensity]), Results[Chain] = "HC",
Results[Result_label] = 0)

VAR unique_drug_loads = FILTER(VALUES(Results[Results_label]),
Results[Result_Deconjugation] > 0)

// Calculate intensity corresponding to heavy chain, exclusing HO
VAR heavy_int = CALCULATE( SUM(Results[RawFileResults.Intensity]), Results[Chain] = "HC",
Results[Result_Label] > 0)

VAR heavy_total =
    SUMX(
        unique_drug_loads,
        VAR current_drug_load = Results[Result_label]
            VAR avg_intensity =
            CALCULATE(
                SUM(Results[RawFileResults.Intensity]),
                Results[Result_label] = current_drug_load,
                Results[Chain] = "HC"
```

```
            )
        RETURN
            avg_intensity * current_drug_load
    )

//VAR ratio = 2* (DIVIDE(light_int, light_int + L0_int, BLANK()) + DIVIDE(heavy_total,
heavy_int + H0_int, BLANK()))
VAR ratio = DIVIDE(heavy_total, heavy_int + H0_int, BLANK())

RETURN ratio
```

3.3.1.2. "Deconjugation HC" parameter

```
Deconjugation_HC =
//find DAR for timepoint 0
VAR DAR_H_t0 = CALCULATE([TotalDAR_HC], Results[Time] = "00h")
//Calculate the differnce
VAR DAR_deconj = DAR_H_t0 - [TotalDAR_HC]
RETURN

//return difference between DAR and DAR at t0 as percentage of DAR at t0
DIVIDE(DAR_deconj,DAR_H_t0)*100
```

3.3.2. *Light chain*

3.3.2.1. "TotalDAR LC" parameter

```
//Initialize variables
VAR L0_int = CALCULATE( SUM(Results[RawFileResults.Intensity]), Results[Chain] = "LC",
Results[Result_label] = 0)

// Calculate intensity corresponding to light chain, excluding LO
VAR light_int = CALCULATE( SUM(Results[RawFileResults.Intensity]), Results[Chain] = "LC",
Results[Result_label]  > 0)

VAR ratio = DIVIDE(light_int, light_int + L0_int, BLANK())

RETURN ratio
```

3.3.2.2. "Deconjugation LC" parameter

```
Deconjugation_LC =
//find DAR for timepoint 0
VAR DAR_L_t0 = CALCULATE([TotalDAR_LC], Results[Time] = "00h")
//Calculate the differnce
VAR DAR_deconj = DAR_L_t0 - [TotalDAR_LC]
RETURN

//return difference between DAR and DAR at t0 as percentage of DAR at t0
DIVIDE(DAR_deconj,DAR_L_t0)*100
```
